# Warming Houses

**Published:** 1876-12

**Authors:** 


### WARMING HOUSES.

Good wood burned in fire-places, as in glad days gone by, never to return, is the most healthful of all methods for warming rooms. But the cost of wood renders this use of it impracticable in our large cities at the North.

The next most economical plan is to burn it in stoves. The temperature and quality of the atmosphere of a room heated with wood burned in an old-fashioned ten plate stove, when the thermometer without is hugging zero, compared with the insufficient heat of a common open grate for coal, or the heavy suffocating warmth in furnace heated apartments, is perfectly delightful. The ten plate stove gives a genial warmth, while that from coal is harsh and dry, irritating to the lungs, and giving feverishness to the skin.

Twenty dollars worth of solid sapling oak or hickory wood, at seven dollars a cord, will keep a room of three hundred square feet agreeably warm from the first of October until the first of May. An open grate will require three tons of anthracite coal for the same time at five dollars a ton; but, for a portion of the time, it will not keep a sitting apartment comfortably warm. Half that quantity of coal burned in a good coal stove will be amply sufficient for the same room and time, two thousand pounds, or twenty-five bushels, being a New York ton. Coal evaporates three times as much water as wood, pound for pound, but wood has a great deal of oxygen in it; anthracite coal has none; hence coal consumes the oxygen of the air of a room very rapidly. Forty pounds of coal renders unfit for respiration in twelve hours forty-two thousand gallons of air, all of which, and five times as much more air, is carried up the chimney. No wonder we call it "a draught" up the chimney. Pound for pound, charcoal gives out the most heat, for it is almost pure carbon. But it takes a hundred pounds of wood to make twenty-five pounds of charcoal. Hard coal gives ninety per cent. of heat. Common charcoal gives out a hundred per cent. of heat. Hard coal, stone coal, anthracite coal—all are the same thing—gives out ninety per cent., and wood twenty-five per cent. Soft coal, bituminous coal, such as the Liverpool, Cumberland, and Pittsburgh, gives out from sixty to ninety per cent. of "carbon," which we here use as the synonyme of heat.

What is called "coke" is the charcoal of coal, makes a cheerful fire, and is almost as cleanly as wood to handle.

Peat is half decayed, or rather half fossilized wood—the half-way house between wood and coal. Bituminous or soft coal is still nearer the fossil state, while anthracite coal is the real "stone" coal. The difference between anthracite or hard coal, and bituminous or soft coal is—the latter has not been as long under the influences which convert vegetable matter into coal; whether higher heat or higher pressure, or both, is conjectural. The gas of our dwellings is obtained from soft or bituminous coal; hence hard coal is soft coal without the gas, or the gas having been used up in some other way.

There are two kinds of anthracite coal, red ash and white ash; the latter is used for furnaces and ranges, or cooking purposes, because it does not "clinker"—that is, its cinders do not melt and run together, and thus clog up the furnace and destroy the draught. But for open grates the "Schuylkill peach orchard red ash" coal is best for five reasons:—1. It kindles easier. 2. It burns with a more cheerful blaze. 3. The edges of each ash are smoother, while the edges of the white ash are jagged, "saw-like," so said by those who have examined both with a microscope. 4. It is less dusty than white ash. 5. Thirty pounds of red ash give out as much heat as thirty-six pounds of white ash. The ash of the red is near the color of iron rust; that of the other is more like wood ashes, whitish.

But coal is not always coal. Of two loads of anthracite coal standing side by side, one will yield double the heat given out by the other, and yet nine persons out of ten can perceive no difference—at least they are unable to tell which is the better load. Thus it is that some dealers advertise to sell coal for five, eight, or ten dimes less a ton than other persons, and the poor and the unwisely economical crowd to them, thinking they are getting "great bargains." Such coal would not be taken as a gift by honorable dealers, because they see at once it has "bone" in it—that is, they know that if a scuttle full is burnt, a large per centage, as high as fifty, will be left in the grate in lumps of the color of a burnt bone. Wash it in water, and it is still white, and is wholly useless. Burn fifty pounds of the best coal, and it will leave some five or six pounds of ashes and cinders, while the same amount of advertised "cheap" coal will leave ten or twenty pounds. We have "experimented" in coal, and have a "feeling sense" of its merits. In proportion as a load of coal has broad flat pieces, and of a dull or coal-dust look, it is "bony." If the lumps are of a smooth, shiny black, and in pieces as "broad as they are long"—that is, approaching a "square fracture"—it is the genuine article. The very offer to sell coal fifty cents or more cheaper than the ruling price is suspicious. One of three things is certain—1. Somebody has not got pay for it, and never will. 2. The advertiser is "hard up." 3. Or he is selling an inferior article, and knows it.

Next to a wood fire, the hot water pipe system gives the most genial heat, as it does not consume the oxygen of the air to anything like the extent which must result from the air coming in contact with hot iron. The nearer the heat emanates from the floor the better, not only because warm air naturally ascends, but also because, when a room is heated from the ceiling, the head is kept too warm, and serious ailments ensue.

Ten years the price of this Journal may be saved any family which uses coal largely, by remembering that the quantity of coal is determined as accurately by measurement as by one of Fairbank's best scales. A bin or box of thirty-four and a half feet cubical, holds exactly one ton of two thousand pounds of white ash coal, such as is used in ranges, stoves, and furnaces, but it takes thirty-six cubical feet for one ton or two thousand pounds of red ash coal, such as New Yorkers use, for grates. It is perhaps known to few, that no coal dealer in Gotham ever, by any possibility, sells a lawful ton of coal, although he is very clear of purchasing less than a lawful ton of twenty-two hundred and forty pounds, or twenty-eight bushels, each bushel being eighty pounds. A bin which will hold an honest ton of red ash coal should measure forty feet cubical—that is the internal length, breadth and height of the bin multiplied together—thus, four feet broad, five feet long, two feet deep. A seam of coal four feet thick, spreading over an acre, yields five thousand tons, and will make steam enough to do the work of sixteen hundred men for twenty of the best years of their life.

A seam or block of coal as it is in the earth, measuring one yard each way, yields a ton. And when it is remembered that there are two hundred thousand square miles of coal fields already known in the world, and each square mile yields three million tons, it is perfectly clear that there is coal enough left in the world to keep us all warm, and supply all our other fuel necessities for a million of years, as few families use more than fifteen tons a year, although a good-sized steamship consumes six tons an hour.

